# Plastamination: A Rising Concern for Parkinson's Disease

**DOI:** 10.1002/mds.30253

**Published:** 2025-06-03

**Authors:** Roberto Erro, Cristiano Sorrentino, Paolo Barone

**Affiliations:** ^1^ Department of Medicine, Surgery and Dentistry “Scuola Medica Salernitana” University of Salerno Baronissi Italy; ^2^ IRCCS SynlabSDN Naples Italy

**Keywords:** environment, microplastics, nanoplastics, pollution, α‐synuclein

Parkinson's disease (PD) is the fastest growing neurological disorder with an estimated incidence far exceeding that expected based on increased life expectancy,^1^ suggesting a significant environmental contribution.

The current massive increase in plastic production together with exceptionally low rates of recovery and recycling have led to the accumulation of over six gigatons of plastic waste in all environmental compartments, from the atmosphere to the sea to the soil, including the summit of Mount Everest,^2^ a phenomenon leading some authors to coin the term ‘plastamination’.^3^ This debris is broken down through several processes into secondary micro‐ and nanoplastics (MNPs), with particle sizes defined as <5 mm and <1 μm, respectively, which have been discovered widely in different organism specimens across the food chain.^2^, ^3^ Humans are constantly exposed to MNPs, mainly via ingestion and inhalation, intaking on average 0.1–5 g every week.^4^ Inevitably, MNPs have also been detected in several human specimens including the brain.^2^, ^3^ Therefore, MNPs represent a significant, but invisible, threat to human health, but their contribution to the development of PD has not yet been appreciated.

## 1 MNPs Can Enter the Brain Via Multiple Routes

The commonest route of exposure to MNPs is through diet, as demonstrated by their detection in gastrointestinal tissues and stools.^2^ However, their identification in other tissues has suggested their possible translocation across various barriers in the body, prompting further research to explore whether MNPs could accumulate at far distant sites including the brain. Indeed, after ingestion and absorption, MNPs can enter the brain through the blood–brain barrier (BBB).^5^, ^6^ Accordingly, it has been shown that orally consumed MNPs can accumulate in the brain of mice^7^ and fish^8^ models. In the real world, brain accumulation of MNPs has been documented in wild fish in a contaminated estuary.^9^ Conversely, in one study using the terrestrial model organism *Drosophila melanogaster*, MNPs were not found to accumulate in the brain after dietary exposure.^10^ It should be noted, however, that *Drosophila* has a distinct BBB structure compared with vertebrates, which among other reasons might explain this discrepant finding. Indeed, factors such as their shape, size, and concentration or the length of the exposure influence MNPs’ propagation into the brain.^8^, ^11^, ^12^

Beyond the oral route, accumulation of MNPs in the brain can occur after inhalation as demonstrated in mice models^12^, ^13^ and as suggested by a recent study which demonstrated the presence of MNPs in the olfactory bulb of human brains.^14^ Although the most likely hypothesis is that translocation might have occurred via lymphatic vessels that surround the olfactory axons at the level of the cribriform plate, other possibilities include systemic circulation, crossing the BBB, or via the respiratory pathway through the trigeminal nerve.^14^

Furthermore, MNPs can be absorbed percutaneously,^15^ although it is not known whether this route of exposure is relevant for subsequent brain translocation, and via placental transmission. Documentation of MNPs in several areas of offspring's brains including the striatum after maternal exposure throughout gestation^16^ is another source of concern in humans regarding the fact that exposure might start even before birth. Although some of the aforementioned studies (the methodological aspects of which are summarized in supplemental Table S1) used brain lysates suggesting the possibility that the detected MNPs originated from blood vessels, accumulation of MNPs in decedent human brains has been confirmed in an additional study.^17^ In the two available human studies,^14^, ^17^ multiple approaches for MNP detection were used: (1) the cryo‐cuts method, which preserves the spatial context of MNPs within the tissue, allowing their proximity to anatomical structures such as blood vessels to be observed; and (2) the digestion method, which ensures that MNPs that are deeply embedded in the tissue are not overlooked. Therefore, it is very likely that the detected MNPs do not simply originate from blood vessels. Interestingly, MNP levels were 7–30 times greater in brain samples than in liver or kidney,^17^ which suggests, among other possibilities, that clearance mechanisms might be less efficacious in the brain. Of note, MNP levels were found to be significantly higher in 2024 than in 2016 brain samples,^17^ emphasizing their exponentially rising environmental presence.

MNPs entering the brain via multiple routes can then propagate at different sites. Supporting evidence for this was found in one study showing that inhaled MNPs were transported from the olfactory bulb to the cerebrum, cerebellum, and basal forebrain.^18^

## 2 The Effects of MNPs on the Gut–Brain Axis

Given the ease of access through the oral route and the critical role of the gut−brain axis in brain functioning, the hypothesis that MNPs might further exert their neurotoxic effects indirectly via the gut−brain axis has been raised.^19^ Although there are no investigations that have explored directly the possible gut‐mediated, neurotoxic effects of MNPs on PD pathophysiological pathways and/or in PD animal models, a group of studies have provided evidence of their detrimental results on the gut–brain axis in non‐PD models, reporting outcomes including microbiota alterations, disrupted intestinal barrier permeability, oxidative stress, inflammation, neurotoxicity, neurotransmitter release alterations, and behavioral disturbances.^19^ In this section, we therefore briefly speculate about the possible mechanisms reconnecting these lines of evidence to PD.

Although some inconsistencies exist about the spread of ⍺‐synuclein (⍺‐syn) from the gut to the brain, some evidence has supported the hypothesis that a yet unknown insult would first induce pathology in a peripheral organ, like the gut, to subsequently spread to the brain (eg, in ‘body‐first PD’). Within this framework, cross‐seeding by various amyloidogenic proteins has been demonstrated,^20^ certain amyloid‐producing bacteria have been found enriched in the microbiome of PD patients,^21^ and gut microbial amyloids were shown to increase ⍺‐syn accumulation in *Caenorhabditis elegans*.^22^ Moreover, ⍺‐syn aggregation can be promoted by lipopolysaccharide,^23^ a major component of Gram‐negative bacteria, and exposures to Enterobacteriaceae has been shown to exacerbate ⍺‐syn pathology in both ⍺‐syn‐ and toxic‐based PD models.^24^, ^25^, ^26^ These lines of evidence support the concept that altered microbiota may trigger and/or exacerbate ⍺‐syn misfolding, and given that MNPs have been demonstrated to alter microbiota composition,^19^ this might represent one mechanism linking MNPs’ exposure to PD through the gut–brain axis.

An additional, not mutually exclusive, mechanism linking MNPs to PD is through inflammation. Indeed, MNPs have been shown to disrupt the intestinal barrier permeability and induce local and systemic inflammation.^19^ The latter can be driven by both direct effects on the mucosal cells as well as by change in the microbiota composition and both aspects seem relevant to PD. Indeed, gastrointestinal mucosal inflammatory damage has been estimated to confer a 76% greater risk of developing PD.^27^ The importance of co‐occurrent disruption of the epithelial barrier, inflammation, and PD risk may be further supported by its possible association with inflammatory bowel diseases.^28^ Moreover, microbiota alterations, with a reduction of taxa with anti‐inflammatory effects, have been reported in PD patients.^29^, ^30^ Local inflammation can therefore disrupt microbiota composition, triggering or fostering ⍺‐syn misfolding; and subsequent systemic inflammation might further reverberate to the brain, priming microglial cells into an active state responsible for stronger responses dealing with an incipient neurodegenerative process.^31^ These processes are arguably intertwined and might be reasonably triggered by MNPs in subjects at risk for developing PD.

## 3 The Effects of MNPs on the Dopaminergic System

Preliminary studies have demonstrated changes in social and motor behavior associated with alterations in dopaminergic circuits in rodent^32^ and fish^33^ models exposed to MNPs. In a 28‐day oral toxicity study, MNPs induced gene expression alteration and cell‐specific responses in mouse brains that were primarily linked with energy metabolism disorder and mitochondrial dysfunction in all brain cells, including those of the substantia nigra pars compacta (SNc) and striatum, and that were associated with diminished neurobehavioral and motor activity.^34^ Similarly, in another 28‐day repeated oral gavage study in mice, the open‐field test revealed a dose‐dependent decrease in movement distances for exposed mice.^35^ The behavioral findings were associated with targets and toxicity mechanisms shared in PD‐like neurodegeneration. In fact, staining experiments on nerve cells unveiled a dose‐dependent reduction in Nissl bodies and TH‐positive cells as well as a dose‐dependent elevation in TUNEL‐positive cells indicative of an increase of apoptotic cells in the SNc of mice exposed to MNPs.^35^ Further transcriptomic analysis revealed changes in differentially‐expressed‐genes associated with calcium ion homeostasis, which were upregulated, and with ATP metabolic processes, which were decreased.^35^ Accordingly, there was an elevation in mitochondrial calcium concentration and a reduction in tissue ATP content in the midbrain of exposed mice, which was not observed in the cortex, hippocampus, and striatum.^35^ Another study explored MNP‐induced cytotoxicity, mitochondrial integrity and functioning, and ATP level alteration in dopaminergic‐differentiated SH‐SY5Y cells showing significant mitochondrial damage, characterized by altered morphology, reduced mitochondrial membrane potential, and decreased ATP production followed by excessive mitophagy and subsequent cell death.^36^ Notably, in vivo experiments demonstrated the potential of melatonin of mitigating dopaminergic neuron loss and motor impairments by restoring mitophagy regulation in mice.^36^ Although there are methodological differences across the aforementioned studies (Table S2), they provide converging evidence of the selective vulnerability of midbrain dopaminergic neurons mainly through mitochondrial dysfunction, linking these alterations to reduced motor activity. The molecular mechanisms whereby these alterations occur remain largely unknown, but in one study molecular docking analyses and dynamic simulations suggested that they might result from the interaction of MNPs with the mitochondrial complex I,^36^ and overwhelming evidence suggests that dopaminergic neurons are particularly vulnerable to mitochondrial stressors.^37^

## 4 The Effects of MNPs on α‐Syn Aberrant Folding

Initial evidence has been produced that MNPs, especially anionic polystyrene particles, have high affinity with α‐syn protein through a hydrophobic binding between the benzene ring and the amphipathic domain and the adjoining non‐amyloid component (NAC) domain of α‐syn.^38^, ^39^, ^40^ This interaction induces aromatic acid residue exposure and modifies α‐syn secondary structure, promoting its fibrillization with a decrease in α‐helices monomers and an increase in β‐sheet oligomers, which have even higher affinity with MNPs.^38^, ^39^, ^40^ Furthermore, MNPs speed the aggregation kinetics of the NAC domain in a dose‐dependent manner.^39^ Cell studies have further demonstrated co‐localization of MNPs and α‐syn aggregates at the lysosomal level and a mild lysosomal dysfunction that further slows down aggregated α‐syn degradation and an increase in fibril‐seeded pS129–α‐syn inclusions.^41^ An increase in α‐syn pathology was further observed in animal model studies.^39^, ^40^ Notably, α‐syn pathology could be demonstrated across interconnected brain regions including dopaminergic neurons in the SNc and it was observed in about 30% of wild‐type mice exposed to MNPs only (ie, without α‐syn pre‐fibrils), suggesting a ‘de‐novo’ induction.^40^ The methodological details of these studies are available in Table S3.

## 5 Concluding Remarks

**FIG. 1:**
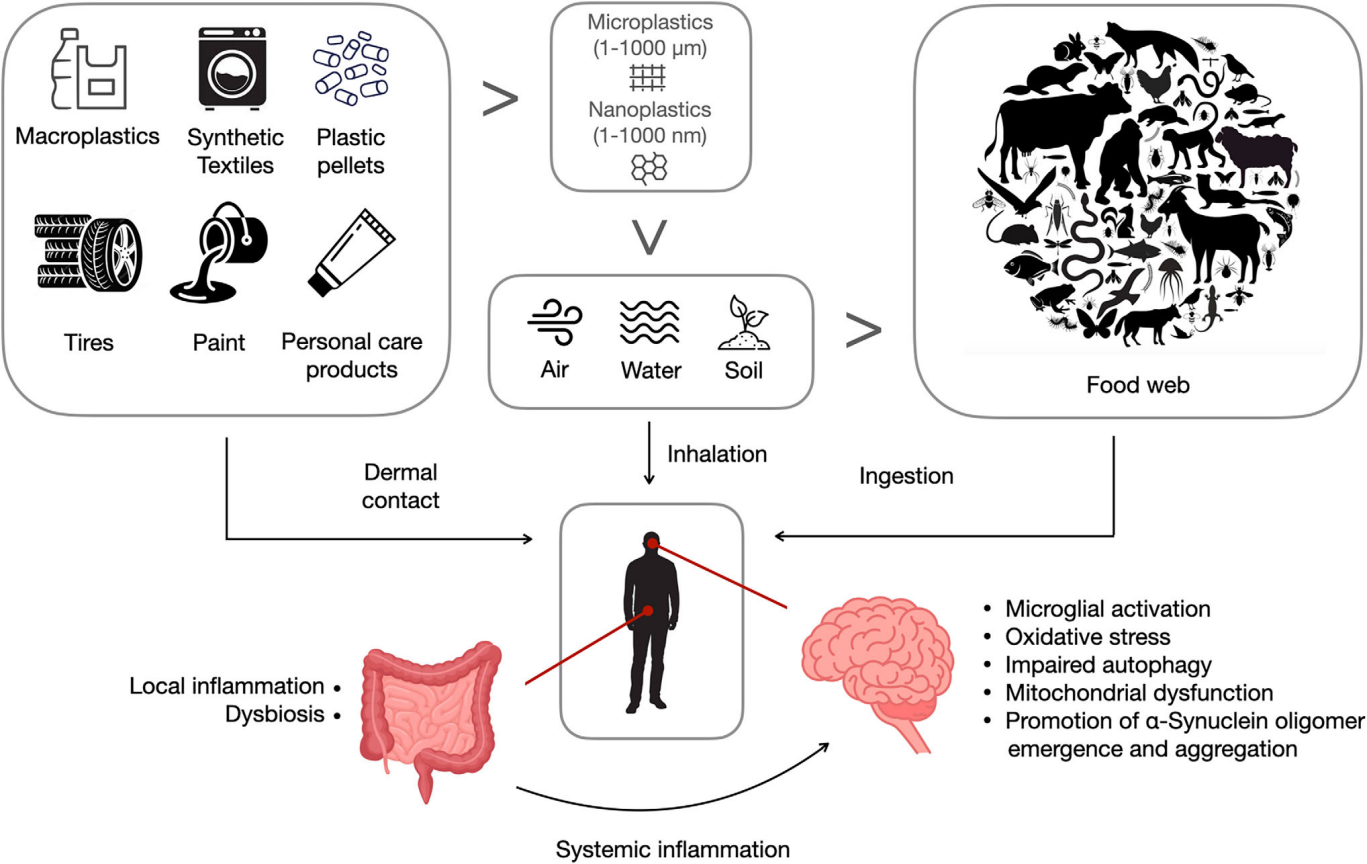
Environmental exposure to plastics and their impact on gut and brain health. [Color figure can be viewed at wileyonlinelibrary.com]

The evidence that MNPs can initiate and/or foster the pathophysiological mechanisms that are the basis of PD are only beginning to appear (Fig. 1) and, in the current viewpoint, we have collated existing research to suggest that MNPs might likely represent a major environmental culprit of this prevalent disorder, which has long been postulated to be a multifactor disease, despite the search for its environmental contributors having been mostly unsuccessful. Clearly, MNPs are appearing to contribute to other neurogenerative diseases, promoting β‐amyloid aggregation peptides in Alzheimer's disease and increasing oxidative stress in amyotrophic lateral sclerosis (ALS), which emphasizes their more general neurotoxic effects.^42^ However, the evidence reviewed here would suggest a selective vulnerability of the dopaminergic system to MNPs as well as their promotion of α‐syn oligomer emergence and fibrillization that would account for the motor deficits observed in animal models. It is therefore conceivable to speculate that MNP exposure might trigger PD pathophysiology in humans in predisposed subjects, according to a double‐ or multi‐hit framework.^43^, ^44^

Since this research is still only in its early stages, there remain several knowledge gaps, especially in terms of methodology, which require immediate attention. First, while we have here collated evidence about MNPs on a broad scale, it has been demonstrated that their shape, size, and aging, among other factors, influence their neurotoxic properties.^8^, ^11^, ^12^ Second, future research should evaluate the neurotoxic effects of MNPs at environmentally realistic concentrations.^42^ Third, most research has explored the effects of single, specific MNPs, but humans are constantly exposed to a multitude of MNPs and other pollutants that are commonly bound to plastics and that exacerbate their neurotoxicity such as plasticizers, flame retardants, mechanical stabilizers, and persistent organic pollutants.^42^ Moreover, MNPs are well known for vector transport of heavy metals, which are arguably another environmental culprit of PD,^45^ and additional evidence is being generated concerning their combined neurotoxic effects.^42^, ^46^ All these gaps clearly add a layer of complexity, but one possible avenue of future research could be exploring the exposome, which is defined as the totality of environmental exposures throughout an individual's life.^47^ Exposomic science currently operates on an omics scale, enabling the simultaneous examination of various molecular components, including genomics, transcriptomics, proteomics, and metabolomics, and allowing for a holistic investigation of the intricate interplay between different environmental influences and biological responses.^47^

Environmental plastamination is predicted to double by 2040 and, given this increasing concern, global action is therefore needed, even based on the precautionary principle, to reduce emissions, whereas future resea should eventually identify measures to mitigate or counteract MNPs’ neurotoxicity.

## Author Roles

(1) Research Project: A. Design, B. Organization, C. Execution; (2) Statistical Analysis: A. Design, B. Execution, C. Review and Critique; (3) Manuscript Preparation: A. Design, B. Writing of the First Draft, C. Review and Editing the Final Manuscript.

R.E.: 3A, 3B, 3C.

C.S.: 3C.

P.B.: 3C.

## Full Financial Disclosures for the Preceding 12 Months

Nothing to report.

## Supporting information


**Data S1.** Supporting Information.

## Data Availability

Data sharing not applicable to this article as no datasets were generated or analysed during the current study.

## References

[1] GBD 2021 Nervous System Disorders Collaborators . Global, regional, and national burden of disorders affecting the nervous system, 1990–2021: a systematic analysis for the global burden of disease study 2021. Lancet Neurol 2024;23(4):344–381. 10.1016/S1474-4422(24)00038-3 38493795 PMC10949203

[2] Thompson RC , Courtene‐Jones W , Boucher J , Pahl S , Raubenheimer K , Koelmans AA . Twenty years of microplastics pollution research‐what have we learned? Science 2024; 386(6720):eadl2746. 10.1126/science.adl2746. 39298564

[3] Santoro A , Marino M , Vandenberg LN , Szychlinska MA , Lamparelli EP , Scalia F , et al. PLASTAMINATION: outcomes on the central nervous system and reproduction. Curr Neuropharmacol 2024;22(11):1870–1898. 10.2174/1570159X22666240216085947. 38549522 PMC11284724

[4] Senathirajah K , Attwood S , Bhagwat G , Carbery M , Wilson S , Palanisami T . Estimation of the mass of microplastics ingested – a pivotal first step towards human health risk assessment. J Hazard Mater 2021;404(Pt B):124004. 10.1016/j.jhazmat.2020.124004 33130380

[5] Kopatz V , Wen K , Kovács T , Keimowitz AS , Pichler V , Widder J , et al. Micro‐ and nanoplastics breach the blood‐brain barrier (BBB): biomolecular corona's role revealed. Nanomaterials (Basel) 2023;13(8):1404. 10.3390/nano13081404. 37110989 PMC10141840

[6] Shan S , Zhang Y , Zhao H , Zeng T , Zhao X . Polystyrene nanoplastics penetrate across the blood‐brain barrier and induce activation of microglia in the brain of mice. Chemosphere 2022;298:134261. 10.1016/j.chemosphere.2022.134261 35302003

[7] Garcia MM , Romero AS , Merkley SD , Meyer‐Hagen JL , Forbes C , Hayek EE , et al. In vivo tissue distribution of polystyrene or mixed polymer microspheres and metabolomic analysis after oral exposure in mice. Environ Health Perspect 2024;132(4):47005. 10.1289/EHP13435 38598326 PMC11005960

[8] Afrose S , Tran TKA , O'Connor W , Pannerselvan L , Carbery M , Fielder S , et al. Organ‐specific distribution and size‐dependent toxicity of polystyrene nanoplastics in Australian bass (Macquaria novemaculeata). Environ Pollut 2024;341:122996. 10.1016/j.envpol.2023.122996 37995956

[9] Barboza LGA , Otero XL , Fernández EV , Vieira LR , Fernandes JO , Cunha SC , et al. Are microplastics contributing to pollution‐induced neurotoxicity? A pilot study with wild fish in a real scenario. Heliyon 2023;9(1):e13070. 10.1016/j.heliyon.2023.e13070 36711285 PMC9880392

[10] Yan W , Li ZJ , Lin ZY , Ji SQ , Tse WKF , Meng ZQ , et al. Microplastic exposure disturbs sleep structure, reduces lifespan, and decreases ovary size in *Drosophila melanogaster* . Zool Res 2024;45(4):805–820. 10.24272/j.issn.2095-8137.2024.038 38894523 PMC11298679

[11] Xiong F , Liu J , Xu K , Huang J , Wang D , Li F , et al. Microplastics induce neurotoxicity in aquatic animals at environmentally realistic concentrations: a meta‐analysis. Environ Pollut 2023;318:120939. 10.1016/j.envpol.2022.120939 36581239

[12] Vojnits K , de León A , Rathore H , Liao S , Zhao M , Gibon J , et al. ROS‐dependent degeneration of human neurons induced by environmentally relevant levels of micro‐ and nanoplastics of diverse shapes and forms. J Hazard Mater 2024;469:134017. 10.1016/j.jhazmat.2024.134017 38518696

[13] Shanmugiah J , Zaheer J , Im C , Kang CM , Kim JS . Comparison of PET tracing and biodistribution between ^64^Cu‐labeled micro‐and nano‐polystyrene in a murine inhalation model. Part Fibre Toxicol 2024;21(1):2. 10.1186/s12989-023-00561-7 38297341 PMC10829228

[14] Amato‐Lourenço LF , Dantas KC , Júnior GR , Paes VR , Ando RA , de Oliveira Freitas R , et al. Microplastics in the olfactory bulb of the human brain. JAMA Netw Open 2024;7(9):e2440018. 10.1001/jamanetworkopen.2024.40018 e2440018 39283733 PMC11406405

[15] Akpojevwe Abafe O , Harrad S , Abou‐Elwafa AM . Assessment of human dermal absorption of flame retardant additives in polyethylene and polypropylene microplastics using 3D human skin equivalent models. Environ Int 2024;186:108635. 10.1016/j.envint.2024.108635 38631261

[16] Zhang Y , Tian L , Chen J , Liu X , Li K , Liu H , et al. Selective bioaccumulation of polystyrene nanoplastics in fetal rat brain and damage to myelin development. Ecotoxicol Environ Saf 2024;278:116393. 10.1016/j.ecoenv.2024.116393 38714083

[17] Nihart AJ , Garcia MA , El Hayek E , Liu R , Olewine M , Kingston JD , et al. Bioaccumulation of microplastics in decedent human brains. Nat Med 2025;31(4):1114–1119. 39901044 10.1038/s41591-024-03453-1 PMC12003191

[18] Liu X , Zhao Y , Dou J , Hou Q , Cheng J , Jiang X . Bioeffects of inhaled nanoplastics on neurons and alteration of animal behaviors through deposition in the brain. Nano Lett 2022;22(3):1091–1099. 10.1021/acs.nanolett.1c04184 35089039

[19] Grodzicki W , Dziendzikowska K , Gromadzka‐Ostrowska J , Kruszewski M . Nanoplastic impact on the gut‐brain axis: current knowledge and future directions. Int J Mol Sci 2021;22(23):12795. 34884598 10.3390/ijms222312795 PMC8657997

[20] Friedland RP , Chapman MR . The role of microbial amyloid in neurodegeneration. PLoS Pathog 2017;13(12):e1006654. 29267402 10.1371/journal.ppat.1006654 PMC5739464

[21] Scheperjans F , Aho V , Pereira PA , Koskinen K , Paulin L , Pekkonen E , et al. Gut microbiota are related to Parkinson's disease and clinical phenotype. Mov Disord 2015;30(3):350–358. 25476529 10.1002/mds.26069

[22] Fernandez‐Calvet A , Matilla‐Cuenca L , Izco M , Navarro S , Serrano M , Ventura S , et al. Gut microbiota produces biofilm‐associated amyloids with potential for neurodegeneration. Nat Commun 2024;15(1):4150. 38755164 10.1038/s41467-024-48309-x PMC11099085

[23] Monteiro Neto JR , Lima VA , Follmer C . Fibrillation of α‐synuclein triggered by bacterial endotoxin and lipid vesicles is modulated by N‐terminal acetylation and familial Parkinson's disease mutations. FEBS J 2024;291(6):1151–1167. 38069536 10.1111/febs.17027

[24] Wang C , Lau CY , Ma F , Zheng C . Genome‐wide screen identifies curli amyloid fibril as a bacterial component promoting host neurodegeneration. Proc Natl Acad Sci U S A 2021;118:34. 10.1073/pnas.2106504118 PMC8403922 34413194

[25] Schmit KJ , Garcia P , Sciortino A , Aho VTE , Pardo Rodriguez B , Thomas MH , et al. Fiber deprivation and microbiome‐borne curli shift gut bacterial populations and accelerate disease in a mouse model of Parkinson's disease. Cell Rep 2023;42(9):113071. 37676767 10.1016/j.celrep.2023.113071 PMC10548091

[26] Sampson TR , Challis C , Jain N , Moiseyenko A , Ladinsky MS , Shastri GG , et al. A gut bacterial amyloid promotes alpha‐synuclein aggregation and motor impairment in mice. elife 2020;9:9. 10.7554/eLife.53111 PMC7012599 32043464

[27] Chang JJ , Kulkarni S , Pasricha TS . Upper gastrointestinal mucosal damage and subsequent risk of Parkinson disease. JAMA Netw Open 2024;7(9):e2431949. 39235810 10.1001/jamanetworkopen.2024.31949 PMC11378005

[28] Freuer D , Meisinger C . Association between inflammatory bowel disease and Parkinson's disease: a mendelian randomization study. NPJ Parkinsons Dis 2022;8(1):55. 35534507 10.1038/s41531-022-00318-7 PMC9085764

[29] Huang B , Chau SWH , Liu Y , Chan JWY , Wang J , Ma SL , et al. Gut microbiome dysbiosis across early Parkinson's disease, REM sleep behavior disorder and their first‐degree relatives. Nat Commun 2023;14(1):2501. 37130861 10.1038/s41467-023-38248-4 PMC10154387

[30] Krueger ME , Boles JS , Simon ZD , Alvarez SD , McFarland NR , Okun MS , et al. Comparative analysis of Parkinson's and inflammatory bowel disease gut microbiomes reveals shared butyrate‐producing bacteria depletion. NPJ Parkinsons Dis 2025;11(1):50. 10.1038/s41531-025-00894-4. 40108151 PMC11923181

[31] Forloni G , La Vitola P , Cerovic M , Balducci C . Inflammation and Parkinson's disease pathogenesis: mechanisms and therapeutic insight. Prog Mol Biol Transl Sci 2021;177:175–202. 33453941 10.1016/bs.pmbts.2020.11.001

[32] Kim NH , Choo HI , Lee YA . Effect of nanoplastic intake on the dopamine system during the development of male mice. Neuroscience 2024;555:11–22. 10.1016/j.neuroscience.2024.07.018 39033990

[33] Hoyo‐Alvarez E , Arechavala‐Lopez P , Jiménez‐García M , Solomando A , Alomar C , Sureda A , et al. Effects of pollutants and microplastics ingestion on oxidative stress and monoaminergic activity of seabream brains. Aquat Toxicol 2022;242:106048. 10.1016/j.aquatox.2021.106048 34875488

[34] Liang B , Huang Y , Zhong Y , Li Z , Ye R , Wang B , et al. Brain single‐nucleus transcriptomics highlights that polystyrene nanoplastics potentially induce Parkinson's disease‐like neurodegeneration by causing energy metabolism disorders in mice. J Hazard Mater 2022;430:128459. 10.1016/j.jhazmat.2022.128459 35739658

[35] Liang B , Deng Y , Zhong Y , Chen X , Huang Y , Li Z , et al. Gastrointestinal incomplete degradation exacerbates neurotoxic effects of PLA microplastics via oligomer nanoplastics formation. Adv Sci (Weinh). 2024;11(28):e2401009. 10.1002/advs.202401009. 38751156 PMC11267364

[36] Huang Y , Liang B , Li Z , Zhong Y , Wang B , Zhang B , et al. Polystyrene nanoplastic exposure induces excessive mitophagy by activating AMPK/ULK1 pathway in differentiated SH‐SY5Y cells and dopaminergic neurons in vivo. Part Fibre Toxicol 2023;20(1):44. 10.1186/s12989-023-00556-4. 37993864 PMC10664492

[37] Haddad D , Nakamura K . Understanding the susceptibility of dopamine neurons to mitochondrial stressors in Parkinson's disease. FEBS Lett 2015;589(24 Pt A):3702–3713. 10.1016/j.febslet.2015.10.021 26526613 PMC4679488

[38] Ghosal S , Bag S , Bhowmik S . Insights into the binding interactions between microplastics and human α‐synuclein protein by multispectroscopic investigations and amyloidogenic oligomer formation. J Phys Chem Lett 2024;15(25):6560–6567. 10.1021/acs.jpclett.4c00731 38885454

[39] Liang X , Andrikopoulos N , Tang H , Wang Y , Ding F , Ke PC . Nanoplastic stimulates the amyloidogenesis of Parkinson's alpha‐synuclein NACore. Small 2024;20(14):e2308753. 10.1002/smll.202308753 37988678 PMC10994764

[40] Liu Z , Sokratian A , Duda AM , Xu E , Stanhope C , Fu A , et al. Anionic nanoplastic contaminants promote Parkinson's disease‐associated α‐synuclein aggregation. Sci Adv 2023;9(46):eadi8716. 10.1126/sciadv.adi8716 37976362 PMC10656074

[41] Jeong A , Park SJ , Lee EJ , Kim KW . Nanoplastics exacerbate Parkinson's disease symptoms in *C. elegans* and human cells. J Hazard Mater 2024;465:133289. 10.1016/j.jhazmat.2023.133289 38157817

[42] Liu S , He Y , Yin J , Zhu Q , Liao C , Jiang G . Neurotoxicities induced by micro/nanoplastics: a review focusing on the risks of neurological diseases. J Hazard Mater 2024;469:134054. 10.1016/j.jhazmat.2024.134054 38503214

[43] Dorsey ER , Bloem BR . Parkinson's disease is predominantly an environmental disease. J Parkinsons Dis 2024;14(3):451–465. 38217613 10.3233/JPD-230357 PMC11091623

[44] Erro R , Bhatia KP , Tinazzi M . Parkinsonism following neuroleptic exposure: a double‐hit hypothesis? Mov Disord 2015;30(6):780–785. 25801826 10.1002/mds.26209

[45] Vellingiri B , Suriyanarayanan A , Abraham KS , Venkatesan D , Iyer M , Raj N , et al. Influence of heavy metals in Parkinson's disease: an overview. J Neurol 2022;269(11):5798–5811. 35900586 10.1007/s00415-022-11282-w

[46] Yu J , Chen L , Wu B . Size‐specific effects of microplastics and lead on zebrafish. Chemosphere 2023;337:139383. 10.1016/j.chemosphere.2023.139383 37394195

[47] Sakowski SA , Koubek EJ , Chen KS , Goutman SA , Feldman EL . Role of the exposome in neurodegenerative disease: recent insights and future directions. Ann Neurol 2024;95:635–652. 38411261 10.1002/ana.26897 PMC11023772

