# Supplementary material for: Plastamination: A Rising Concern for Parkinson's Disease
Source: Mov Disord. 2025 Jun 3;40(8):1528–33. doi: 10.1002/mds.30253 (PMC12371614; doi:10.1002/mds.30253)
Supplement: Supplementary file 1 — Data S1. Supporting Information. [file MDS-40-1528-s001.pdf]

Plastamination: a rising concern for Parkinson's Disease – Supplemental Material

Abbreviations

AchE: acetylcholinesterase; AD: Alzheimer's Disease; BBB: blood-brain barrier; ALS: amyotrophic lateral sclerosis; DA: dopamine; DOPA: dihydroxyphenylalanine; DOPAC: 3,4-dihydroxyphenilacetic acid; DLB: dementia with Lewy Body; HD: Huntington's Disease; HVA: homovanillic acid; MNPs: micro-nanoplastics; MP: microparticles; MRI: magnetic resonance imaging; NA: not applicable/available; NP: nanoparticles; PE: polyethylene; PLA: Polylactic acid; PS: polystyrene; PD: Parkinson's Disease; PVC: polyvinylchloride; ROS: reactive oxygen species; SNc: substantia nigra pars compacta; TH: tyrosine hydroxylase; w/v: weight in volume; 5-HIAA: 5-hydroxy-3-indoleaceticacid; 5-HT: serotonin; 5-HTP: 5-hydroxytryptophan.

Table S1. Details of the studies supporting MNPs accumulation in the brain

First author, year, reference	Details on the micro- or nano-plastic used in the study	Administration route	Length of exposure	Dosage	Model	Methods of detection
Kopatz, 2023, [5]	The used particles contained 4 chains of polystyrene, each with 100 styrene monomer units, folded together into a ca. 5 nm nanoparticle.<br>Three different sizes were used (9.55 ± 0.13 µm particles; 1.14 ± 0.03 µm particles; and 0.293 ± 0.008 µm particles) mixed at equal weight concentrations of 0.3 mg/size/dose in sterile water	Oral	2 or 4h	Mice were assigned randomly to 3 treatment groups and either left untreated or gavaged with a single dose of 100 µL of particle mixture.	Male C57Bl/6J mice.	Immunofluorescence staining on 3 µm paraffin-embedded histological brain sections
Shan, 2022, [6]	Nominal diameter of the particles was of 42 nm diluted in 0.1 M phosphate-buffered saline	Oral	7 days	Mice were sorted by body weight and assigned by random number method to one of 5 groups including four NPs groups with different doses (0.5, 2.5, 10, and 50 mg/kg	Male C57BL/6 J mice (8-week-old).	Immunofluorescence on 16 µm coronal slices of brain post-fixed in 4% paraformaldehyde after mice sacrifice. To investigate microglia phagocytosis a staining method with a specific microglia marker (ionized calcium binding

				of body weight) and one control (receiving an equal volume of 0.1 M phosphate-buffered saline).		adaptor molecule 1) was used.
Garcia, 2024, [7]	5µm microspheres of polystyrene or of a mixed plastics treatment in a 1:1:1 ratio consisting of polystyrene, polyethylene, and poly-lactic-co-glycolic acid, in deionized water (10-mg/ml concentration)	Oral	Twice a week over 4 weeks	0 mg/week (control group), 2mg/week and 4mg/week	Male and female C57BL/6 mice (8–12 week-old).	After exposure, mice were euthanized and exsanguinated to ensure removal of blood from major organs. Light microscopy and spectroscopy was used on digested prefrontal cortex samples
Afrose, 2024, [8]	Polystyrene NPs with sizes of 50 nm and 1 µm, each in stock dispersions of 2.5% w/v at volumes of 15 mL and 10 mL,	Oral	2, 3, 4, and 5 days	Three treatment groups (Bass fed with 60 artemia containing NPs of 1 µm or 50 nm or fed with 60 unexposed artemia).	Australian Bass (Macquaria novemaculeata) fed with Artemia exposed to NPs at 20 µg/L for 24h	Immunofluorescence on digested brain samples (no further data available)
Barboza, 2023 [9]	NA (real word study aiming at detecting NPs in brain fishes captured at a contaminated estuary and aimed at being sold for human food consumption)	NA	NA	NA	European seabass (Dicentrarchus labrax Linnaeus), the European flounder (Platichthys flesus Linnaeus) and the flathead grey mullet (Mugil cephalus Linnaeus)	Brain samples (no data available on how they were collected) were filtered through 42 mm diameter glass-microfiber filter membranes (pore size 1.2 µm) and the particles present in the filters were observed through spectroscopy
Vojnits, 2024 [12]	Polystyrene 2 µm MPs	Inhalation	28 days of repeated intranasal exposure (schedule of	two treatment groups: 24 µL phosphate buffered saline (PBS) or 2 µm	C57BL/6 J female mice (8-week-old)	After sacrifice, harvested brains were fixed in liquid nitrogen, or in 4% paraformaldehyde.

			5 days on and 2 days of rest)	MPs (0.68 mg/kg in PBS, representative of 1.2 M particles/day)		30 µm brain sagittal sections were obtained and each section was visually scanned at 10 × magnification with immunofluorescence.
Shanmugiah, 2024 [13]	0.2–0.3-µm sized or 20-nm sized, surface-modified (to allow radiolabeling), polystyrene	Inhalation	Single intratracheal instillation	3 µL MNP solution (concentrated at 2 mg/100 µL) per gram of mouse weight	C57BL/6 J female mice (8-week old)	PET/CT image acquisition in-vivo
Zhang, 2024 [16]	25 nm and 50 nm polystyrene	Oral	17 days	Four concentrations of NP (50, 10, 2.5, and 0.5 mg/kg/day) were utilized.	Healthy pregnant Sprague Dawley rats (to detect transplacental transfer)	Fetal brain samples were fixed in 4% paraformaldehyde and sectioned into 10 µm thick tissue slices with MNPs detected by immunofluorescence
Liu, 2022 [18]	80, 100, 200 nm polystyrene	Inhalation	7 days	Four concentrations of NP (5,10,15, and 20 µg/mL). Total volume of the administered solution not reported	Mice	Immunofluorescence on brain samples dissected after sacrifice (no further details available)

Table S2. Details of the studies demonstrating neurotoxic effect of MNPs onto the dopaminergic system

First author, year, reference	Details on the micro- or nano-plastic used in the study	Administration route	Length of exposure	Dosage	Model	Outcomes	Results
Kim, 2024 [32]	100 nm polystyrene microspheres	Oral	5-6 days to 15 days, depending on the gestation	A solution containing 6.0666×10^{12} particles/kg	Pregnant mice	- Behavioral tests (open-fiedl test; social adaptation	MNPs exposure induced changes in social behavior partly depending on

			time during which pregnant mice were exposed (early gestation to post-natal)	(2.76 mg/kg) was administered daily		test; nest building test)<br>- Electrophysiological tests (local field potentials recorderd from the prefrontal cortex, striatum, nucleus accumbens; amygdala, hippocampus)	developmental stage, and these changes were associated with neural circuits innervated by the dopamine system
Hoyo-Alvarez, 2022, [33]	Low-density Polyethylene particles ranging from 500 to 200 µm (~30% of 500 µm, ~25% of 400 µm, ~25% of 300 µm and ~20% of 200 µm). An additional group was given a MNPs enriched feed exposed to seawater in an anthropogenically impacted area for 2 months in order to enrich the MNPs with the pollutants within the water column.	Oral	90 days [outcomes collected at T90 and and after a posterior detoxification period of 30 days (T120)].	10% MNPs feed. The quantity of feed varied depending on the biomass of the tank (size and number of individuals), normally oscillating between 50-75 g of feed	Gilthead seabream (*Sparus aurata*)	- Oxidative stress biomarkers (enzyme activities of Catalase, Superoxide Dismutase, and glutathione S-transferase; malondialdehyde levels)<br>- Neurotransmitter levels/activity (AchE, DA, NA 5-HT, DOPA 5-HTP; DOPAC, HVA and 5-HIAA)	MNPs and MNPs+pollutants enriched diet increases the activity of some of the oxidative stress biomarkers (e.g. Catalase and glutathione S-transferase) and alterations on dopaminergic and serotonergic system activity. A tendency to recover enzymatic and brain monoaminergic neurotransmitter levels was observed at T120
Liang, 2022, [34]	50 nm polystyrene spherical particles	Oral	28 days	0.25, 2.5, 25, and 250 mg/kg body weight	Male C57BL/6 J mice	- Behavioral tests (open-field test; rotarod test; grip strength test)<br>- BBB permeability assay (Evans blue dye brain content quantification after retro-orbital injection)<br>- Brain MRI<br>- MNPs detection by fluorescence	- Increased BBB permeability as a function of increased MNPs concentration<br>- MNPs were detected throughout the brain<br>- Transcriptomic analysis revealed that PD pathway

						- Nissl staining on hippocampus, SNc and striatum 4 µm slices<br>- TH positive cell count and insoluble α-synuclein positive cell count by immunofluorescence<br>- ATP content on brain lysates<br>- Transcriptomic analysis on different brain regions	was more severely affected (with down regulation of genes related to ATP metabolism and mitochondrial function) as compared to AD, ALS, HD pathways<br>- less Nissl bodies in the SNc and striatum, but not in the hippocampus as well as significant decrease in T2 relaxation time in the SNc but not striatum at higher MNPs concentrations<br>- misfolded p-α-synuclein accumulated in the TH positive cells in the SNc or striata of mice exposed to MNPs<br>- Exposed mice showed diminished motor activity

Liang, 2024, [35]	PLA polymer and oligomers (after in vivo digestion particles with diameters ranging from <100 nm to 200 nm were detected depending on the time of digestion)	Oral	28 days	2.5 or 25 mg kg^{-1} concentrated solution at a dosage of 10 mL kg^{-1} body weight	C57BL/6 J Mice	- Behavioral test (open field test; elevated plus maze; rotarod test)<br>- Fluorescence imaging on brain homogenates for MNPs detection<br>- Nissl and TUNEL staining and TH positive cell count in different brain areas<br>- Transcriptomic analysis on different brain regions	- Detection of PLA particles in different brain areas, but more concentrated at the midbrain, with an uncompromised BBB permeability<br>- dose-dependent decrease in movement distances for exposed mice<br>- dose-dependent decrease in Nissl bodies in the SNc (but not in the cortex, striatum, and hippocampus) as well as in TH-positive cells in the SNc but not striatum, along with an increase in TUNEL-positive cells<br>- transcriptomic analyses revealed a significant upregulation of genes neuronal mitochondrial

							calcium overload only in the midbrain but not in the cortex or striatum
Huang, 2023 [36]	50 nm Polystyrene particles	In vivo experiment: oral	In vitro experiment: 48 hours<br>In vivo experiment: 28 days	In vitro experiment: 0, 0.5, 5, 50 and 500 µg/mL.<br>In vivo experiment: 250 mg/kg/day	In vitro experiment: human SH-SY5Y neuroblastoma cell line differentiated into dopaminergic neurons<br>In vivo experiment: C57BL/6 J mice	In vitro experiment: fluorescence and transmission electron microscopy for MNPs cell uptake; cell viability assay; ROS assay; mitochondrial dysfunction assessment and stress test; molecular docking)<br>In vivo experiment: behavioral tests; Nissl staining on SNc and striatum; anti-TH, anti-voltage-dependent anion channel and anti-LC3 antibodies immunofluorescence	- MNPs detected in differentiated SH-SY5Y cells, including mitochondria, leading to cell toxicity and reduced viability (antioxidant treatment failed to fully rescue viability, suggesting ROS-independent cytotoxicity)<br>- MNPs caused significant mitochondrial damage (altered morphology, reduced membrane potential, decreased ATP and disrupted mitochondrial respiration, through interference with complex I)

							- In vivo experiments demonstrated that melatonin could mitigate dopaminergic neuron loss and motor impairments by restoring mitophagy regulation in mice

Table S3. Details of the studies exploring the effects of MNPs onto α-syn aggregation

First author, year, reference	Details on the micro- or nano-plastic used in the study	Administration route	Length of exposure	Dosage	Model	Outcomes/Techniques	Results
Ghosal, 2024, [38]	PS-MNP (100 nm)<br>PVC-MNP (100 nm)<br>PE-MNPs (34-50 µm)	NA	NA	Increasing MNPs concentration (0-100 µg/mL) with a fixed human α-syn concentration (50 or 100 µg/mL)	Wild-type human α-syn expressed in E.coli	Several multi-spectroscopic techniques, such as Fourier transform infrared, circular dichroism, and ultraviolet-visible spectroscopy	The amide bond microenvironment in the α-syn protein was altered in the presence of all MNPs , resulting in more aromatic acid residues and reduction of the content of α-helices and increase of that of β -sheets (this effect was particularly evident with PS-MNPs) with higher effects at higher MNPs concentrations. The study of the α-syn oligomers revealed a higher percentage of β-

							sheet/extended structure upon MNPs exposure.
Liang, 2024, [39]	PS-MNPs (50 nm)	NA	NA	20 and 200 µg/mL	Molecular experiments: α-syn NACore peptides<br>Cell experiments: HMC3 cells<br>Animal experiments: Zebrafish	Molecular experiments: Electrospray ionization time-of-flight mass spectrometry; Negative-stain transmission electron microscopy (TEM); binding assays using dynamic light scattering (DLS); molecular dynamic (MD) simulations.<br>Cell experiments: oxidative stress<br>Animal experiments: hatching, survival, and development	NAC monomers are rapidly absorbed by MNPs, likely due to hydrophobic interactions. Faster aggregation kinetics of the NACore peptide in a MNPs dose-dependent manner, compared to NACore alone. MNPs promoted NACore fibrillization (wider and more ribbon-like NACore fibrils and sheets), especially at higher concentrations (200 µg/mL).<br>MNPs at both concentrations altered distributions of NACore's charge states at 20 min post incubation. At 48h post incubation, reduced population of NACore dimers indicated peptide conversion from oligomers to amyloid fibrils. In silico simulations suggest that the binding can be attributed to hydrophobic interactions between the NACore sidechains and the MNPs benzene

							rings, resulting in an enrichment of large complex with oligomers possessing β-sheets. MNPs exposure increased oxidate stress and late apoptosis elicited by NACore in the microglial HMC3 (from 7.74% to 15.24% cells) An impairment to zebrafish hatching, survival, and development was observed in vivo upon their embryonic exposure to NAcore and MNPs.
Liu, 2023, [40]	PS-MNPs with ~39.5±0.7 nm average diameter	NA	Variable (up to 7 days)	High concentration of α-syn monomeric protein (i.e., ~1 mg per mL) with 1 nM MNPs. Thus, the stoichiometry was ~1 nanoplastic particle for every 70,000 monomer α-synuclein particles (i.e., 0.007% w/v plastic in solution).	- Wild-type human α-synuclein<br>- mature hippocampal neurons in culture<br>- Non-transgenic outbred CD1 mice<br>- Frontal brain samples from patients with DLB	Negative-stain transmission electron microscopy (TEM); binding assays using dynamic light scattering (DLS); real-time quaking induced conversion (RTQuIC) assays; molecular dynamic (MD) simulations; surface plasmon resonance	Presence of multiple α-syn fibrils detectable with TEM as early as 3 days after exposure. One nanoplastic particle can form a stable complex with more than 100 α-syn monomers within minutes (DLS) sub-nanomolar concentrations of nanoplastic can nucleate the formation of α-syn fibrils (RTQuIC), with anionic MNPs accelerating new

							fibril growth. Molecular dynamic simulations showed a stable α-syn and anionic MNPs complex characterized by strong electrostatic attraction and compaction of the amphipathic domain and the adjoining non-amyloid component (NAC) domain (truncated α-synuclein proteins at the amphipathic domain failed to interact with the anionic MNPs). The strength of interaction, estimated with the use of surface plasmon resonance, revealed higher affinity with aggregated α-syn (i.e. fibrils). In cultured neurons, MNPs are internalized by a clathrin-mediated mechanism, co-localize at the lysosomes with α-syn aggregates, induce a mild lysosomal dysfunction and attenuate α-syn degradation as early as 12h post exposure. A modest concentration (1 nM) of MNPs increases pS129-α-syn

							pathology as compared to lower concentrations. Strital co-injection of MNPs (15 μg) with α-syn fibrils results in a broadening of nanoplastic distribution similar to α-synuclein fibril particles without MNPs, with ~20% of dopaminergic neurons in the SNpc 3 days post injection being positive for both α-syn fibrils and MNPs in their cell soma (75% of signal co-localization). Co-injections further results in the formation of mature cytoplasmic pS129-α-syn inclusions in dopaminergic neurons and a large increase across the cortical mantle and amygdala (which is not observed with α-syn fibrils injection alone). Interestingly, 30% of mice injected with MNPs alone showed pS129-α-syn pathology, suggesting "de-novo" induction of pathology. Frontal cortex brain tissues from Lewy

							body dementia with strong α-synuclein seeding activity also yield strong styrene ion traces
Jeong, 2024, [41]	25 nm polystyrene particles with different surface groups, including pristine particles, COOH-particles and NH2-particles	Both worms and cells were exposed with MNPs beads	C. elegans: 24 hours or chronic exposure (5 to 7 days) A53T α-syn-SH-SY5Y cells: 24 hours	For C. elegans, MNP beads were diluted with complete K-medium (at 10, 100, or 1000 μg/L.<br><br>For cell experiment, MNP beads were diluted with phosphate buffered saline at 15 mg/L	- C. elegans (including both wild type strains and PD-model strains)<br>- A53T α-syn-SH-SY5Y cells	C. elegans experiments: body length, survival assay, brood size assay, thrashing assay, intestinal barrier function assay, mitochondrial fragmentation in muscle dopaminergic neuronal morphology, α-syn aggregation assay<br>- A53T α-syn-SH-SY5Y cells experiments: α-syn aggregation assay	C. elegans: for short term exposure there was a significant lower growth and locomotor activity at lower concentrations (10 μg/L). Effects on locomotor activity were pronounced for longer exposure of 7, but not 5 days. Highest head bioaccumulation was observed with long exposure (7 days) COOH-MNPs, whereas NH-2 particles mostly induced altered intestinal barrier functional and mitochondrial fragmentation in muscle cells, which correlated with the diminished locomotor activity. In the PD-models, MNPs exposure accelerated degeneration of dopaminergic neurons in the head and higher α-syn aggregate number. These models also had diminished

							locomotor activity upon MNPs exposure.<br>A53T α-syn-SH-SY5Y cells: pristine MNPs induced ~50% increase in α-syn aggregate number.
